# Supplementary figures and images for: Intranasal nanoemulsion adjuvanted S-2P vaccine demonstrates protection in hamsters and induces systemic, cell-mediated and mucosal immunity in mice
Source: PLoS One. 2022 Nov 2;17(11):e0272594. doi: 10.1371/journal.pone.0272594 (PMC9629544; doi:10.1371/journal.pone.0272594)

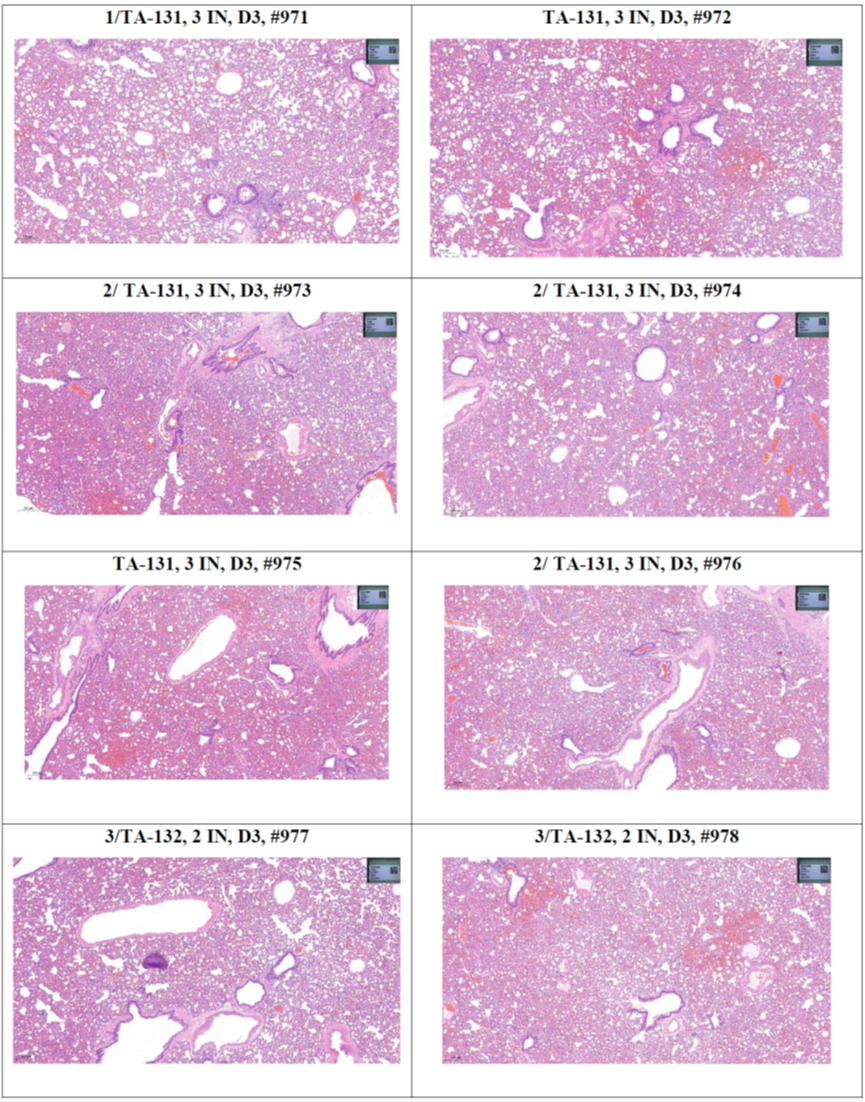

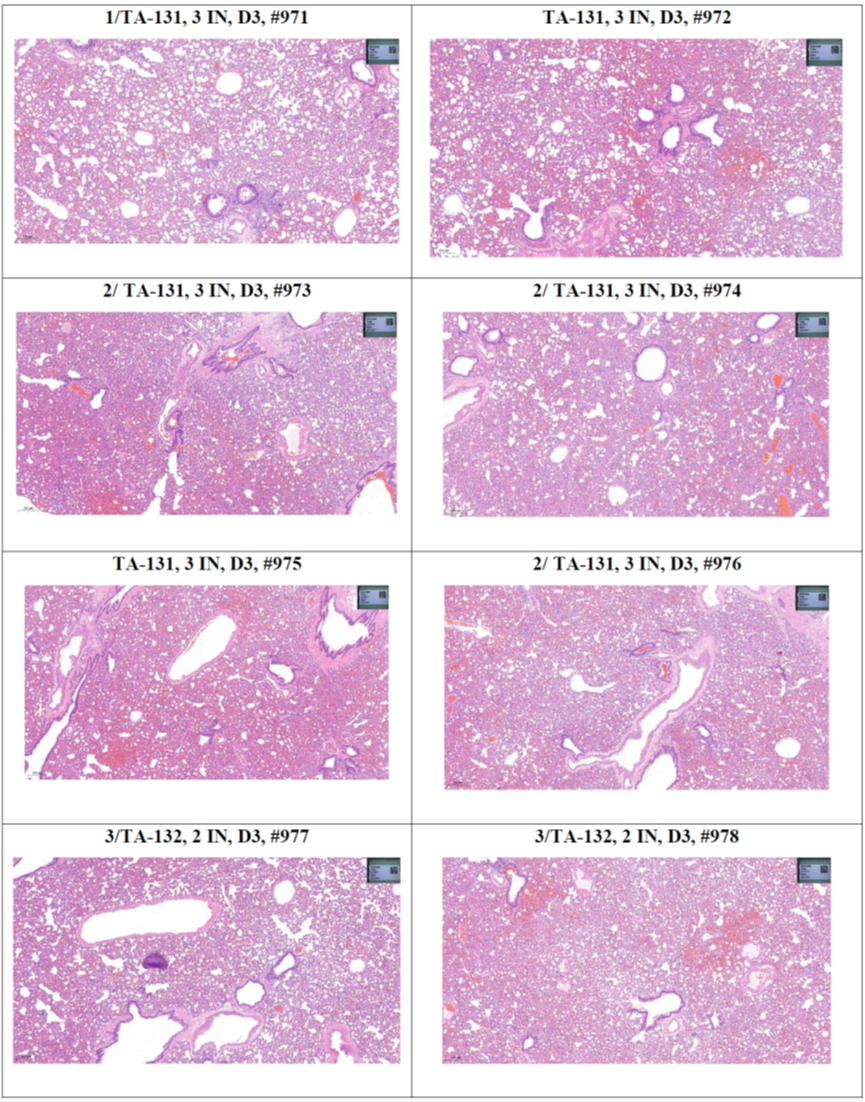


**3 dpi 6 dpi**


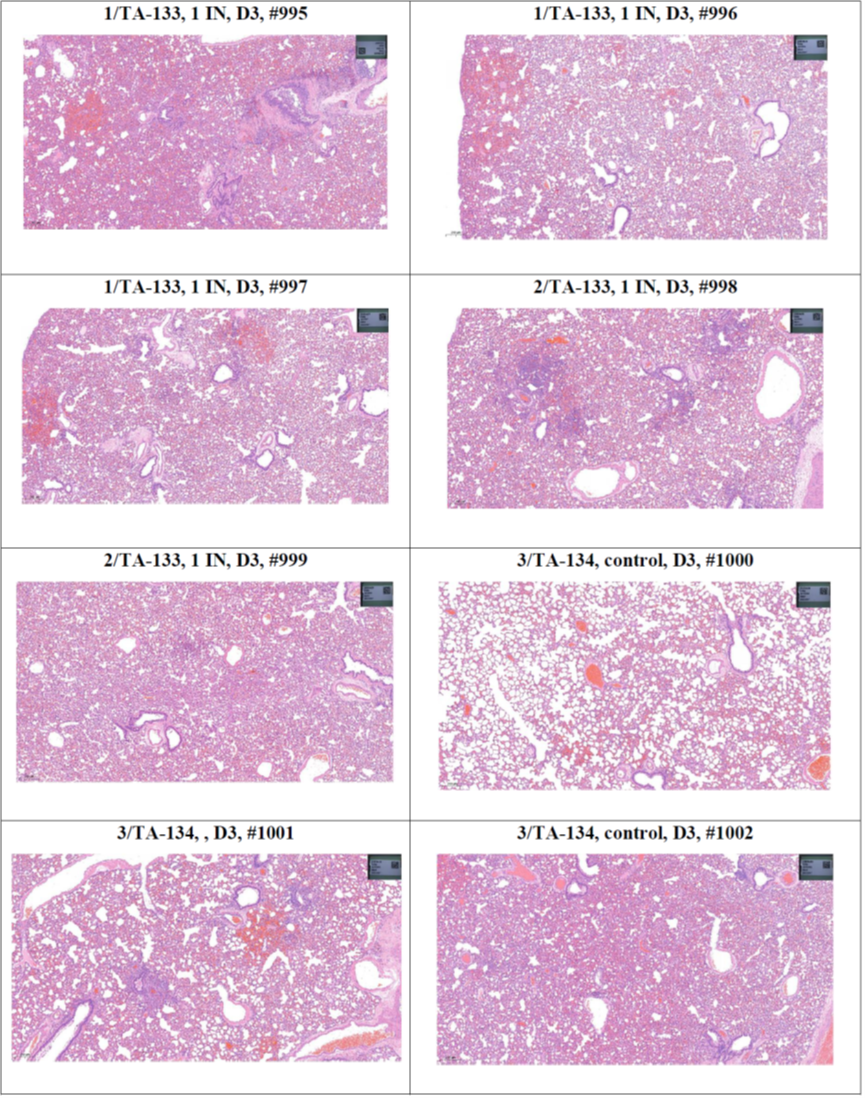

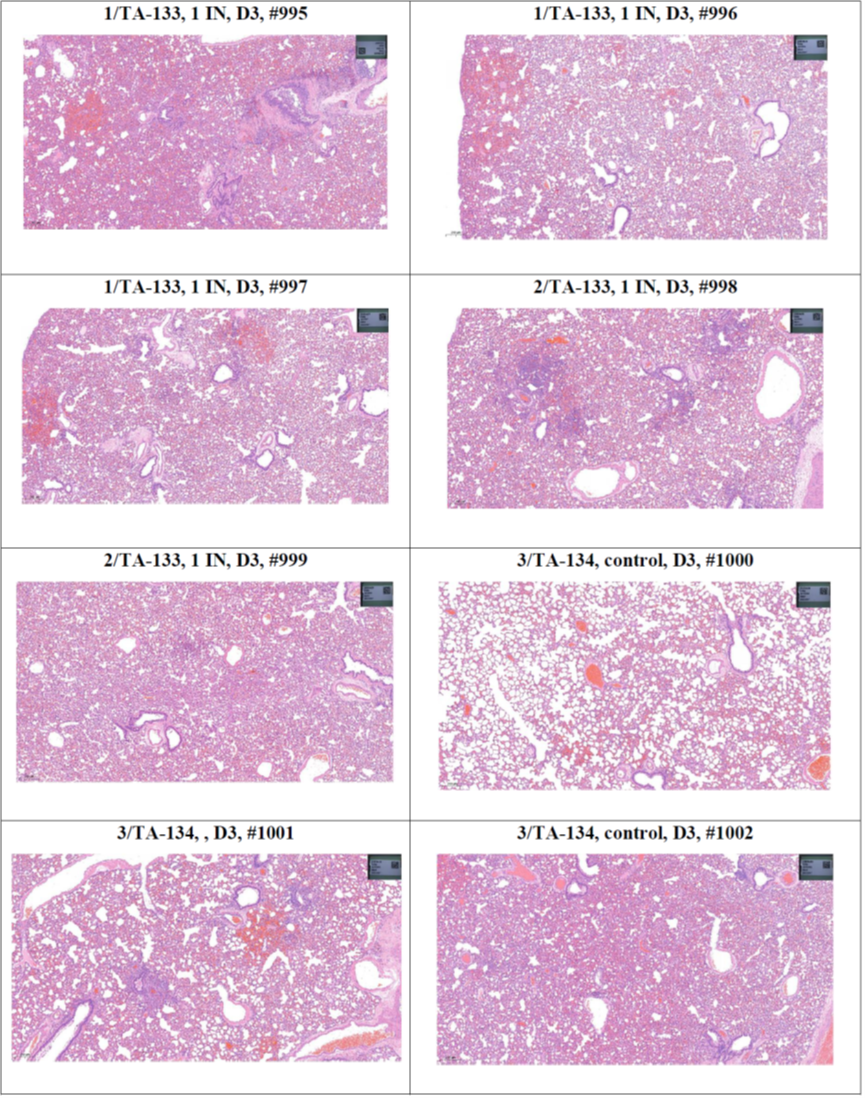

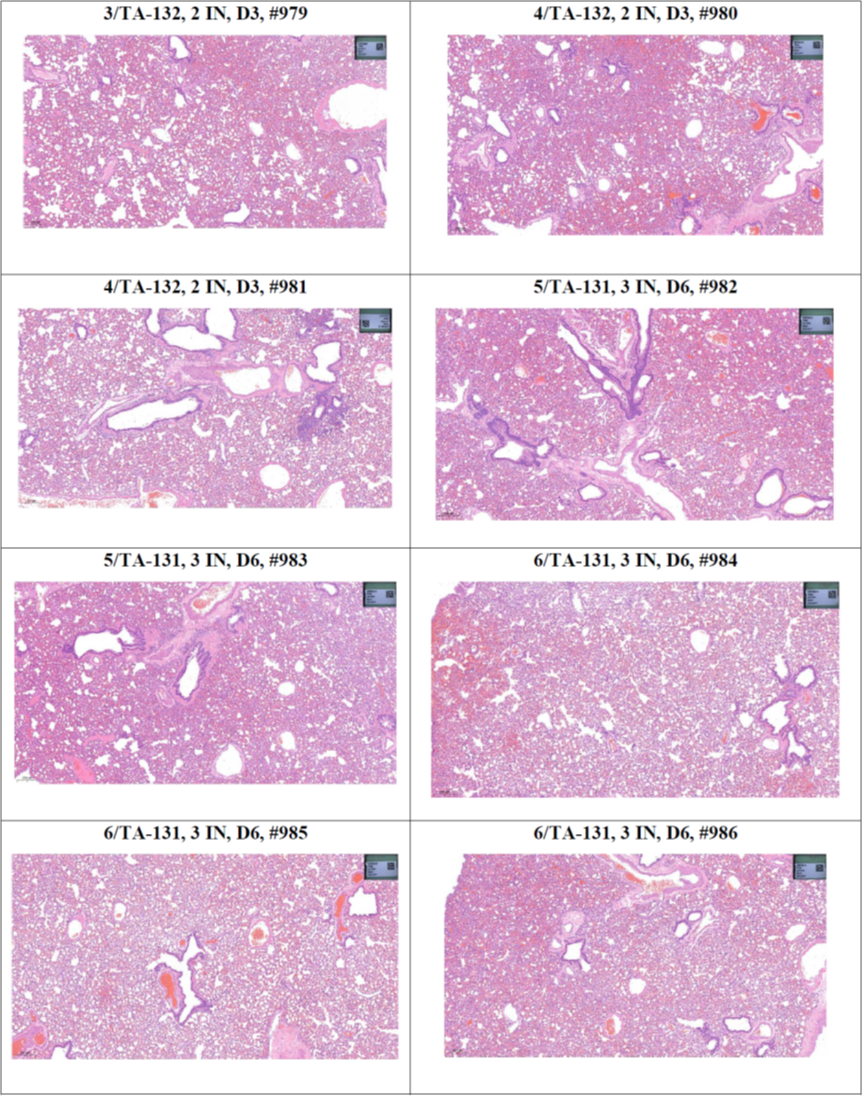

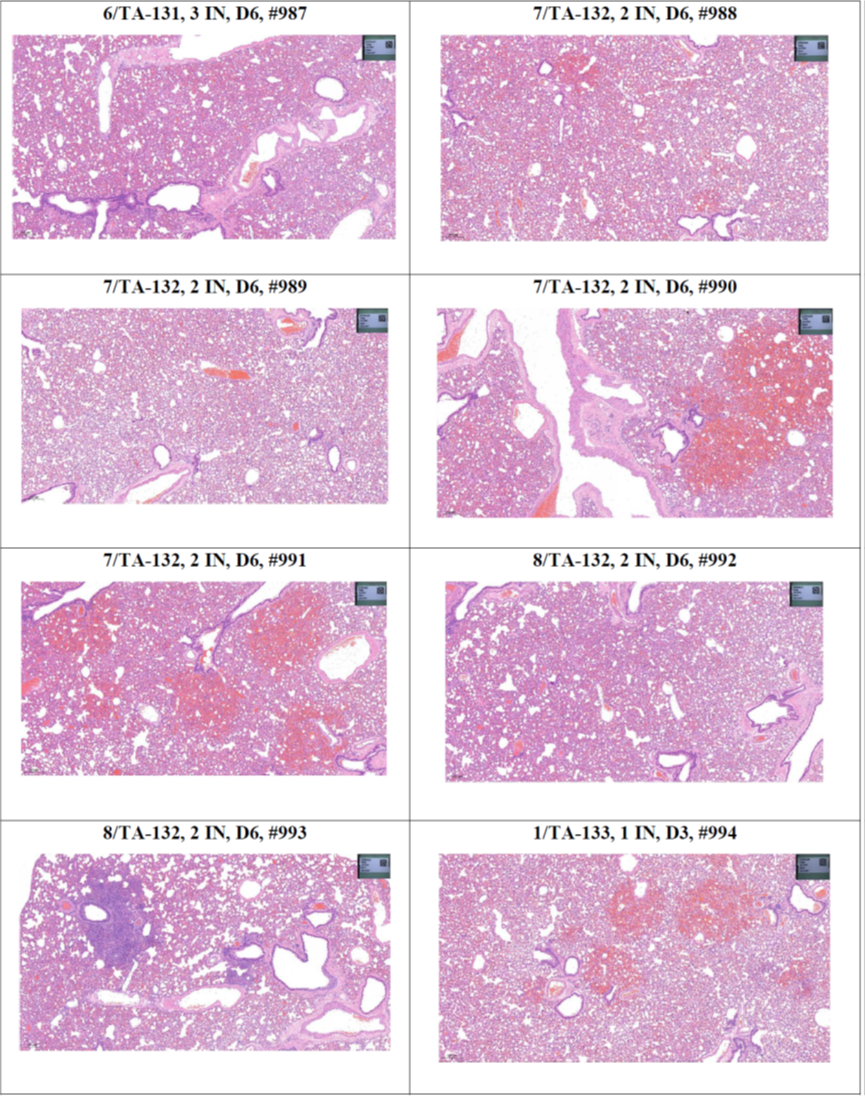

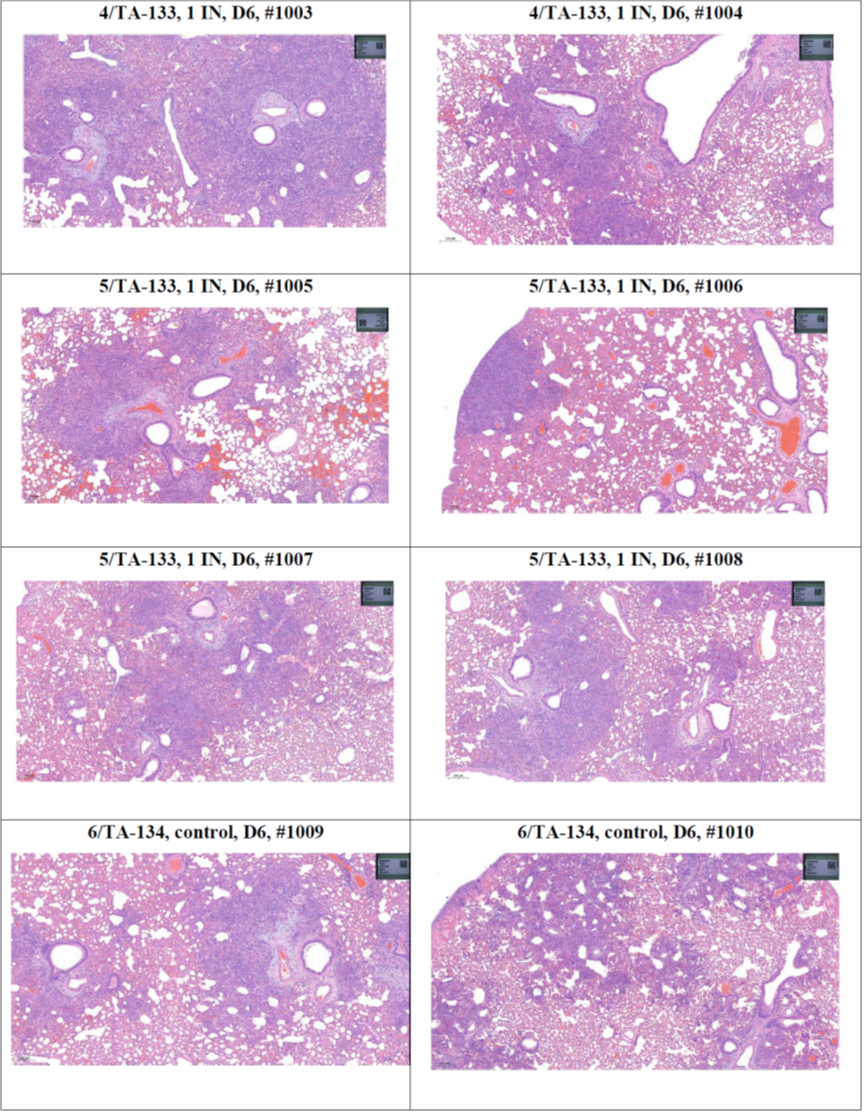

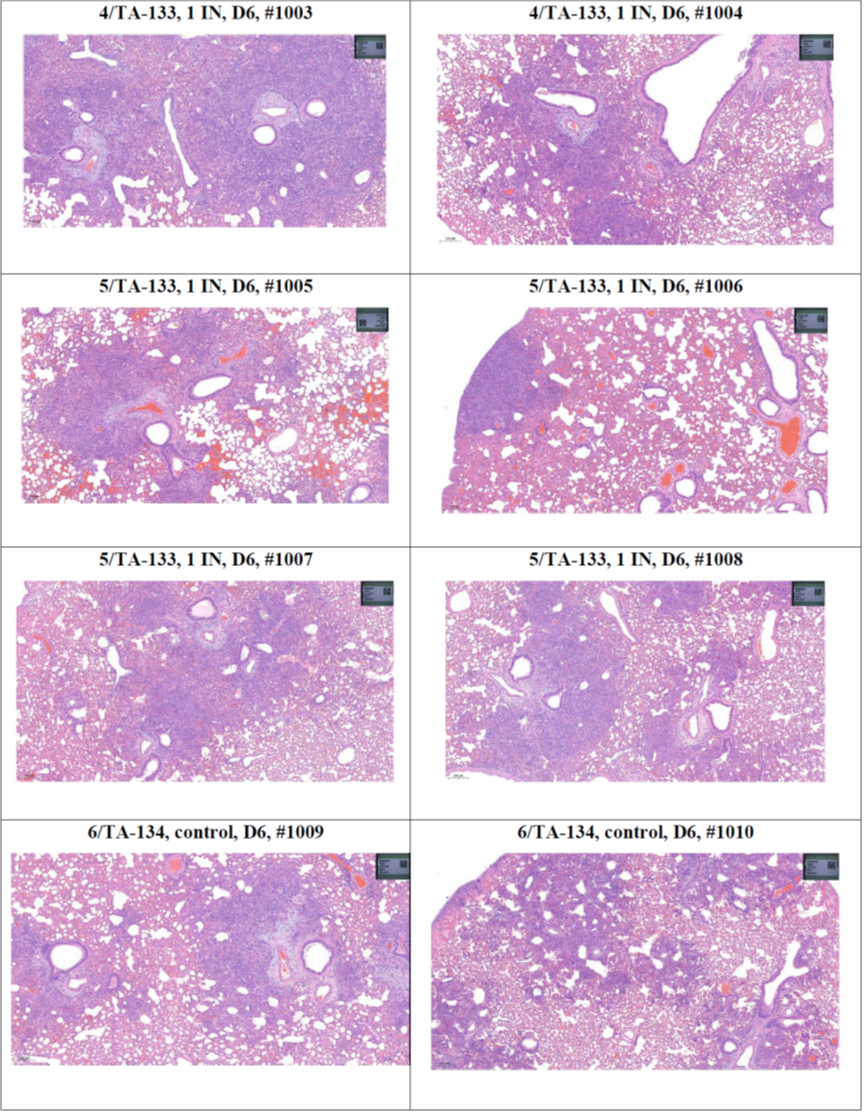


**S-2P/NE01 (x3)**

**S-2P/NE01 (x2)**

**S-2P/NE01 (x1)**

**Naive**

Supplement: S1 Fig — Hamsters were euthanized 3- and 6- days post challenge with SARS-CoV-2 and left lobes were isolated and fixed in 4% paraformaldehyde for one week, sectioned and stained with Hematoxylin and Eosin for scoring. (DOCX) [file pone.0272594.s001.docx]
